# Respiratory *Chlamydia* Infection Induce Release of Hepoxilin A_3_ and Histamine Production by Airway Neutrophils

**DOI:** 10.3389/fimmu.2018.02357

**Published:** 2018-10-15

**Authors:** Katir K. Patel, Wilmore C. Webley

**Affiliations:** Department of Microbiology, University of Massachusetts Amherst, Amherst, MA, United States

**Keywords:** *Chlamydia pneumoniae*, asthma, exacerbation, neutrophils, hepoxilin, histamine release

## Abstract

**Background:** Hepoxilins are biologically active metabolites of arachidonic acid that are formed through the 12-lipoxygenase pathway. Hepoxilin A_3_ is now known to be an important regulator of mucosal inflammation in response to infection by bacterial pathogens and was recently identified as a potent neutrophil chemoattractant in the intestinal mucosa. Our goal in this study was to determine if airway infection with *Chlamydia* in a murine model of allergic airway disease (AAD) induces hepoxilin secretion along with airway neutrophilia.

**Methods:** We utilized an AAD adult Balb/c mouse model to evaluate airway pathology and immune response by assaying bronchoalveolar lavage (BAL) fluid cytokine, cellularity, histidine decarboxylase (HDC) as well as histamine released in response to *in-vivo* chlamydial antigen stimulation of purified airway neutrophils. Hepoxilin A_3_ production was determined by Western blot identification of 12-lipoxygenase precursor (12-LO).

**Results:** Chlamydial infection induced increased production of IL-2, IL-12, TNF-α, and IFN-γ in BAL fluid compared to uninfected animals. *Chlamydia*-infected mice responded with robust airway neutrophil infiltration and upon induction of AAD increased their production of IL-4, IL-5, and IL-13 by >3 fold compared to unsensitized groups. In addition, 12-LO mRNA was upregulated in infected, but not in uninfected AAD mice, suggesting the production of hepoxilin A_3_. mRNA expression of HDC was induced only in neutrophils from the airways of *Chlamydia*-infected mice, but was not seen in AAD only or uninfected controls. When purified neutrophils from infected animals were challenged with chlamydial antigen *in vitro* there was significant histamine release.

**Conclusions:** Our data confirms the production and release of hepoxilin A_3_ in the murine airways concomitant with airway neutrophilia in response to chlamydial infection. We further confirmed that *Chlamydia* provokes the production and release of histamine by these neutrophils. These findings suggest that neutrophils, provoked by *Chlamydia* infection can synthesize and release histamine, thereby contributing directly to airway inflammation.

## Introduction

Asthma continues to be a major public health problem worldwide, and has increased in prevalence considerably over the past three decades(1). The disease is characterized by intermittent airflow obstruction, which develops on a background of chronic inflammation of the airways, driven by CD4^+^ T-lymphocytes, and episodes of acute inflammation, typically dominated by eosinophils (2). Airway eosinophilia is well-described in asthma (3, 4), and currently available anti-inflammatory treatments for asthma almost exclusively target eosinophilic airway inflammation and IgE secretion. However, increased attention is focusing on the clinical heterogeneity of asthma and the argument that failure to recognize and diagnose the underlying mechanisms of different asthma subtypes is a major limitation to progress in asthma control (5).

Numerous molecular and immune system pathways have been implicated in different manifestations of a disease once considered uniform and well-understood. Asthma may be generally characterized into eosinophilic and non-eosinophilic phenotypes based on inflammatory cell patterns in airway secretions. It has been determined that almost 50% of all asthma patients present with non-atopic, non-IgE-dependent, and non-eosinophilic inflammation (6). These patients fail to satisfactorily respond to standard corticosteroid therapy, and this type of steroid-resistant, severe asthma has been linked to the presence of neutrophilic lung inflammation (7–9). Neutrophilic inflammation has also been described in sudden-onset fatal asthma and neutrophil numbers are highly elevated in status asthmaticus, an acute exacerbation of asthma that does not respond well to standard treatments with bronchodilators and inhaled corticosteroids (10). Recent research has confirmed that neutrophil recruitment into the lung tissues is mediated in part by interleukin 17 (IL-17)–producing helper T (T_H_17) cells and neutrophilic inflammation is strongly associated with sudden-onset fatal asthma (11). Since neutrophils are often recruited to the site of an allergic reaction early in the inflammatory process, they may influence clinical presentation and play a role in development of severe chronic asthma and the onset of severe attacks.

*Chlamydia* organisms are prevalent in the lungs of pediatric and adult patients with severe, chronic respiratory disease, and could significantly impact asthma outcomes (12, 13). The pathophysiological features of allergic asthma, including airway remodeling, are thought to result from the expansion of CD4^+^ T cells producing predominantly Th_2_ cytokines, interleukin-4 (IL-4), IL-5, and IL-13. Our lab has also recently demonstrated the significant role of *Chlamydia*-specific IgE antibodies and neutrophils in the pathophysiology of infectious asthma through histamine degranulation and AAD (14, 15). While our research confirms the presence if IL-8 and IL-17 in the *Chlamydia*-infected airways, it was not clear if other neutrophil chemoattractants could also be playing a role here. Neutrophils are among the first responders in innate immunity, rapidly deployed to sites of infection where they efficiently phagocytize and eliminate a variety of pathogens through their robust oxidative burst. However, in cases of chronic disease when there is no timely resolution, neutrophils can exacerbate pathology, promoting, and prolonging asthma symptoms (16, 17). In order to do this, neutrophils must first exit the circulatory system of the lung, navigate through the extracellular milieu, and ultimately cross the mucosal epithelial barrier to reach the bronchoalveolar air space (18, 19).

Hepoxilins are biologically active hydroxyepoxy eicosatrienoic acid metabolites of arachidonic acid, formed through the 12-lipoxygenase pathways (20). Phospholipase A_2_ is the enzyme mainly responsible for the conversion of membrane phospholipids into arachidonic acid, which serves as the precursor for eicosanoids such as hepoxilin A_3_. Hepoxilin A_3_ is a potent neutrophil chemoattractant, produced primarily by pathogen-infected epithelial cells that is believed to facilitate neutrophil migration across mucosal barriers. McCormick et al, have demonstrated that bacterial infections at epithelial surfaces, such as those that line the gut and the lung stimulate the migration of neutrophils through the actions of chemoattractants secreted from epithelial cells that have been pathogen-stimulated (18). The investigators further confirmed that hepoxilin A3 specifically mediates neutrophil migration across the intestinal mucosa (18, 21, 22). These findings implicate hepoxilin A3 as an important inflammatory mediator at mucosal surfaces and could be a potential therapeutic target. We therefore hypothesized that neutrophils, provoked by *C. pneumoniae-*induced lung infection, transmigrated across the respiratory mucosa in partial response to hepoxilin, greatly expanding their capacity to synthesize histamine, thereby contributing to airway inflammation and pathology. We set out to specifically determine if *Chlamydia* infection of the airways induced the release of hepoxilins and if these neutrophils produced histamine in response to airway infection. Our findings confirm the production of significant amounts of hepoxilin A3 in the lungs as well as histamine-producing neutrophils in response to Chlamydia airway infection.

## Methods

### Animals and treatment groups

We obtained IACUC approval through the University of Massachusetts animal care and use committee to utilize animals for this study and we complied with and worked closely with the animal care staff to complete the study. We obtained 2-week-old BALB/c mice from The Jackson Laboratory (Bar Harbor, ME) and kept them in an animal biosafety level II facility for the duration of the study. Animals were divided into four groups of fifteen as follows: Group 1 mice were infected via the airways with *Chlamydia muridarum* [MoPn, approximately 200 inclusion-forming units (IFU) in 50 μl sucrose phosphate-glutamate buffer, SPG] for 14 days before being sensitized and challenged with the 45 kDa egg-white glycoprotein, ovalbumin (OVA) intraperitoneally followed by several challenges via aerosolization; group 2 were mock-infected using the MoPn vehicle, sucrose phosphate glutamate buffer (SPG), challenged and sensitized with OVA on the same schedule as group 1. Note that MoPn has been used historically as a robust representative respiratory model for *C. pneumoniae*. **Group 3** was infected, but challenged and sensitized using saline instead of Ova; **group 4** was mock-infected and mock challenged with saline. BAL fluid was collected from animals and the extent of hepoxilin release evaluated via measurement of hepA3 at days 7, 14, 28, and 42 when animals were sacrificed. Five animals from each group were sacrificed at each time point to obtain BAL fluid, cells and tissue. BALB/c mice infected intranasally with *Chlamydia* (MoPn) and challenged with ovalbumin, as well as uninfected and unchallenged controls were weighed daily and serum was collected weekly. Each tested group had at least 5 animals.

### Ova AAD model

Two weeks post-infection/placebo treatment, mice were sensitized to Ova by intra-peritoneal (IP) injection of 50 μg Ova in 200 μl of 0.9% sterile saline. Seven days after sensitization (day 21), mice were challenged intranasally (IN) with Ova (10 μg in 50 μl sterile PBS, for 3 consecutive days). One day later (day 4 post Ova challenge), control mice were euthanized by sodium pentobarbital overdose and cellular features of acute airway disease (AAD) were characterized. Control mice were mock sensitized and challenged on the same schedule with the sterile saline vehicle.

### Analysis of mouse BAL fluid cellularity and the presence of hepoxilin

Bronchoalveolar lavage fluid (BAL) was obtained by cannulation of the trachea and lavaging the airways with 2 × 1 ml sterile saline. BAL cell counts were performed with a cell counting chamber (hemocytometer, improved Neubauer) under phase microscopy with results expressed as “number of cells per cubic millimeter. BAL differential counts were performed using Wright stained cytospin preparations of BAL. At least 200 cells were counted per slide to obtain statistically significant counts. In order to determine the presence and quantity of hepoxilin in the lung tissue of mice, we utilized Western blots analysis. The presence of 5-LO and 12-LO protein was determined by running lung homogenates on NuPAGE gels (Life Technologies Corporation, Grand Island, NY), transferring to PVDF membrane and probing the blots with anti-5-LO and anti-12-LO antibodies (Santa Cruz Biotech, Dallas, Texas).

### RT PCR

Lung tissue used for RNA extraction was flash frozen in liquid nitrogen or on dry ice. In order to confirm the production of hepA3, messenger RNA was isolated from lung tissue using Trizol reagent extraction according to the manufacturer's instructions (ThermoFisher Scientific). RT-PCR was used to determine expression of mRNA for 5-lipoxygenase (5-LO) 12-lipoxygenase (12-LO), and histidine decarboxylase (HDC), using previously published primers (22).

### Cytokine analysis

Total cytokines secreted in the lung milieu was determined through analysis of BAL fluid. Concentrations of IL-2, IL-4, IL-5, IL-6, IL-10, IL-12, IL-13, IL-17A, IL-23, TNF-α, TGFβ1, and IFN-γ were determined using Multi-Analyte ELISArray Kits (Mouse Th1/Th2/Th17 Cytokines from SABiosciences a QIAGEN company, Valencia CA) according to manufacturer's instructions.

### Isolation of BAL neutrophils and detection of histidine decarboxylase (HDC) mRNA

Cells were isolated from the BAL of mice as described above and utilized for measurement of overall white blood cell infiltration and neutrophil population. Neutrophil isolation from BAL was accomplished with a Mouse Neutrophil Negative Selection Kit (STEMCELL Technologies, Vancouver, BC) according to the manufacturer's instructions. We utilized molecular genetics to determine if neutrophils in *Chlamydia-* infected airways produced and released histamine. Neutrophils were also collected from mice that had not been previously exposed to *Chlamydia*. Following neutrophil isolation, RNA extraction was performed using Trizol extraction (Life Technologies, Grand Island, NY). Expression of HDC mRNA was determined by RT-PCR using previously published primers (23).

### Tissue culture/histamine release

BAL neutrophils were isolated using a mouse neutrophil negative selection kit from STEMCELL Technologies Inc. (Vancouver, Canada). Purified cells were placed into tissue culture plates and stimulated with purified, heat inactivated, chlamydial EBs over a period of 75 min. The supernatant was subsequently analyzed for histamine release using a modified ELISA procedure at 15, 30, 45, 60, and 75 min post-stimulation.

### Serum and BAL antibodies

Serum antibody titer to *Chlamydia* as well as ovalbumin was evaluated in blood collected from tail bleeds of mice at weekly intervals and at time of euthanasia. *Chlamydia* antibody titers were evaluated by enzyme linked Immunosorbent assay (ELISA). The ELISA wells were coated with 100 μl of purified chlamydial EBs (300 μg/ml) and serial dilutions of each mouse serum added. Following the required washes, bound primary antibodies were detected by an AP-conjugated goat anti-mouse secondary antibody (West Grove, PA). Quantitative assessment of OVA-IgE in the BAL fluid and serum was conducted using an OVA-specific mouse-IgE ELISA kit from BD Biosciences, San Jose, CA, according to the manufacturer's instructions. *Chlamydia-*specific IgE antibodies were detected using Western blot assay as previously described (14, 15).

### Statistic

Mice were evaluated in groups of 4. Results are presented as mean± SEM from each test and control group of mice. All analyses were performed using Graph Pad Software and Microsoft Excel. Significant associations or differences are based on the two tailed *T*-test and statistical significance is indicated with *p* < 0.05.

## Results

### Chlamydia lung infection and animal pathology

**Figure 1:**
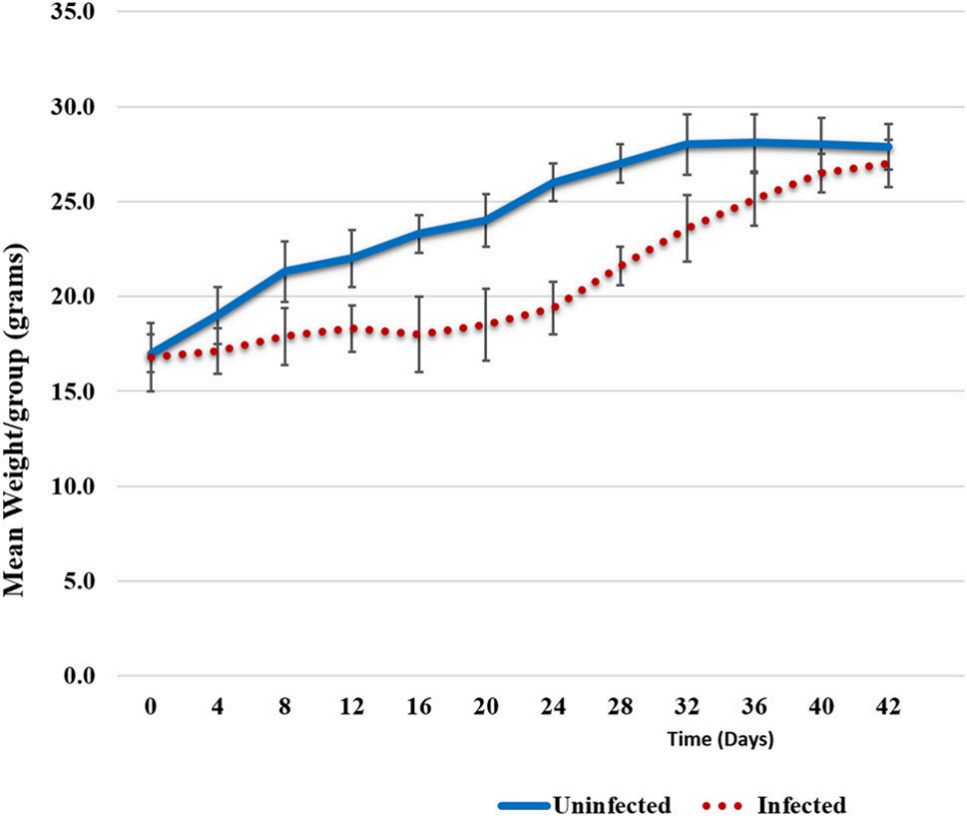
Weight trends. Weight trend graphs show mean weight loss in *Chlamydia* infected animals vs. uninfected animals. The loss can be seen between days 6 and 32 post-infection; on average 5.7 g smaller (30%). However, infected mice recovered lost weight by day 42. Error bars represent variation from the mean weight within each group.

**Figure 2:**
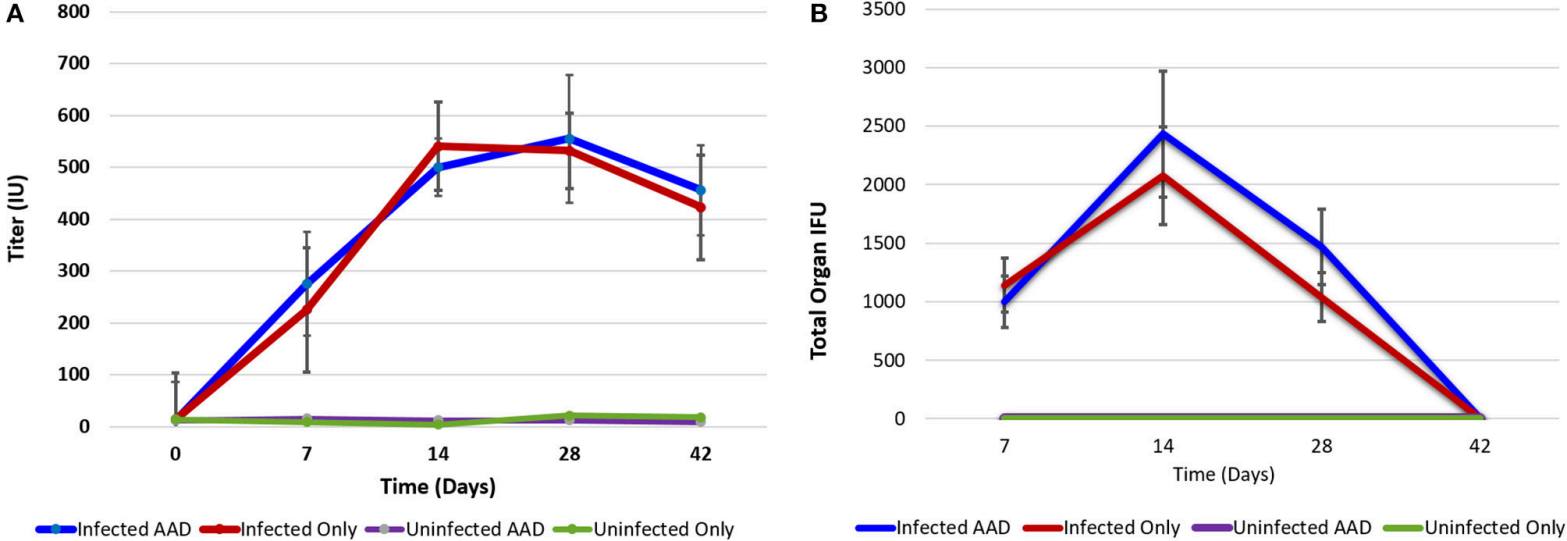
Chlamydia antibody titer and lung bacterial load over time. Antibody titer graph shows strong antibody responses to *Chlamydia* from infected animals but no antibody production in uninfected groups. Infected animals titers to *Chlamydia* began decreasing once infections cleared **(A)**. Chlamydial carriage in the lung peaked 14 days pi **(B)**. Chlamydial organisms were not isolated from lung tissues at 42 days post-infection.

Animals were weighed each day and the weight trend shows significant weight loss in *Chlamydia*-infected vs. uninfected mice starting at 4 days post-infection. The most significant differences in weight gain were observed between days 16 and 32, where on average infected mice were 30% (5.7 g) smaller than uninfected mice. However, the rate of weight gain between infected and uninfected adult groups was not significantly different during the later parts of the time course (Figure 1). To assess the humoral immune response to intranasal chlamydial challenge, sera was collected from each animal and assayed for anti-*Chlamydia* antibody titers every seven days. The data revealed a robust antibody response to *Chlamydia* infection in these mice (Figure 2A). Titers began to decrease in infected animals coincident with the time of infection clearance (28–42 days pi). The concentration of chlamydial organisms in the lungs (IFU/mg) of infected animals increased significantly with time and there was almost a 2-fold increase from day 7 to 14 (Figure 2B). However, total lung carriage began to decline after day 14 and infected animals ultimately cleared the infection by 42 days post-infection.

### WBC and cytokine response to respiratory chlamydial infection

**Figure 3:**
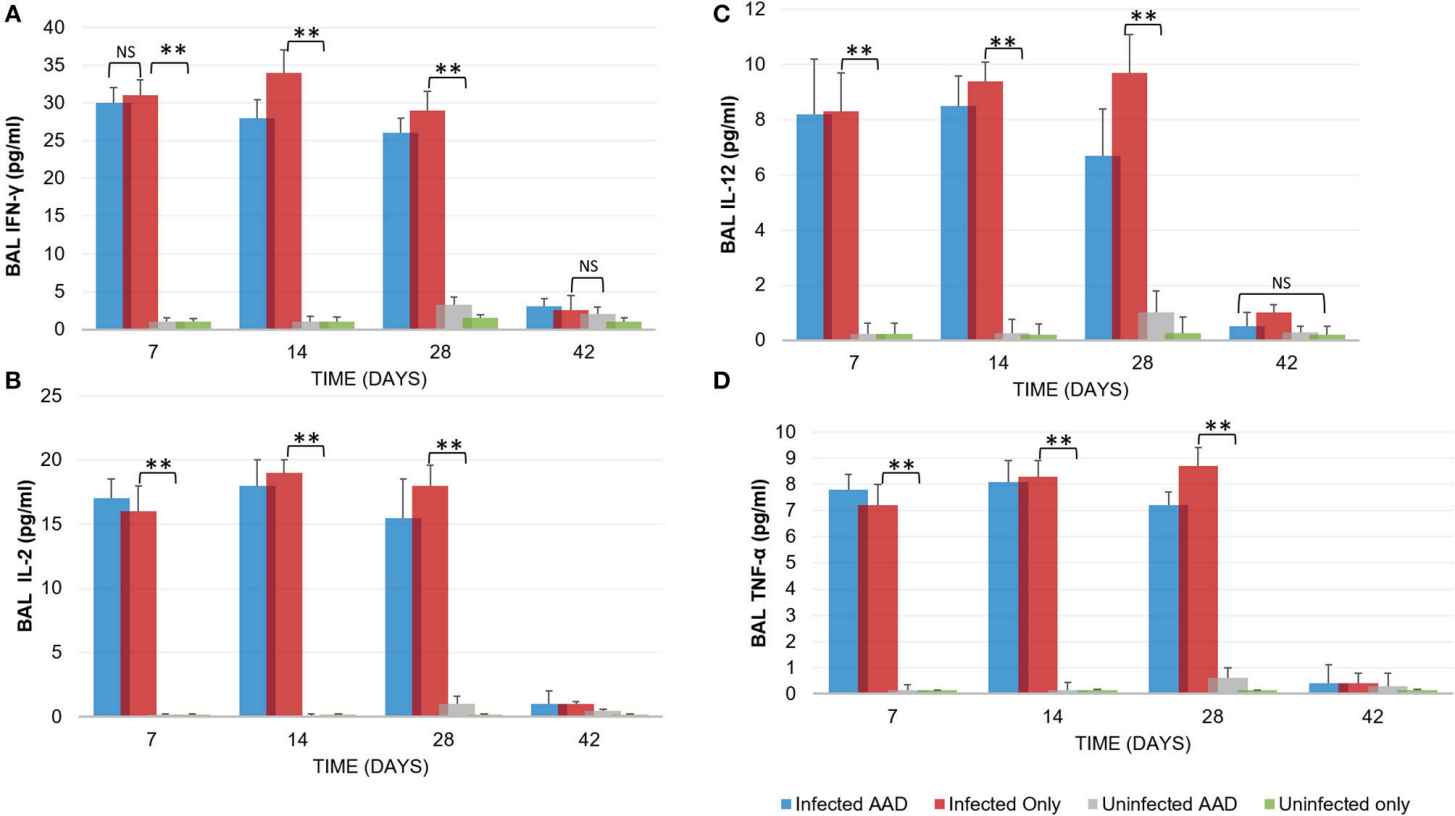
Th1 cytokine levels during chlamydial infection and AAD induction. Infected animals displayed a robust Th1 cytokine response (IFN-γ, IL-2, IL-12, TNF-α, **(A–D)**, respectively; *N* = 5 animals per group) in response to chlamydial infection. Infected mice cleared the infection after 28 days. Uninfected and AAD induced animals did not produce Th1 cytokines. Cytokine production was significantly reduced by day 42, coinciding with clearance of infections. **P < 0.0001 for uninfected controls compared to infected animals; NS is *p* = not significant. There was no significant difference in cytokine levels between infected only and infected AAD animals. Results are representative of 2 independent experiments.

**Figure 4:**
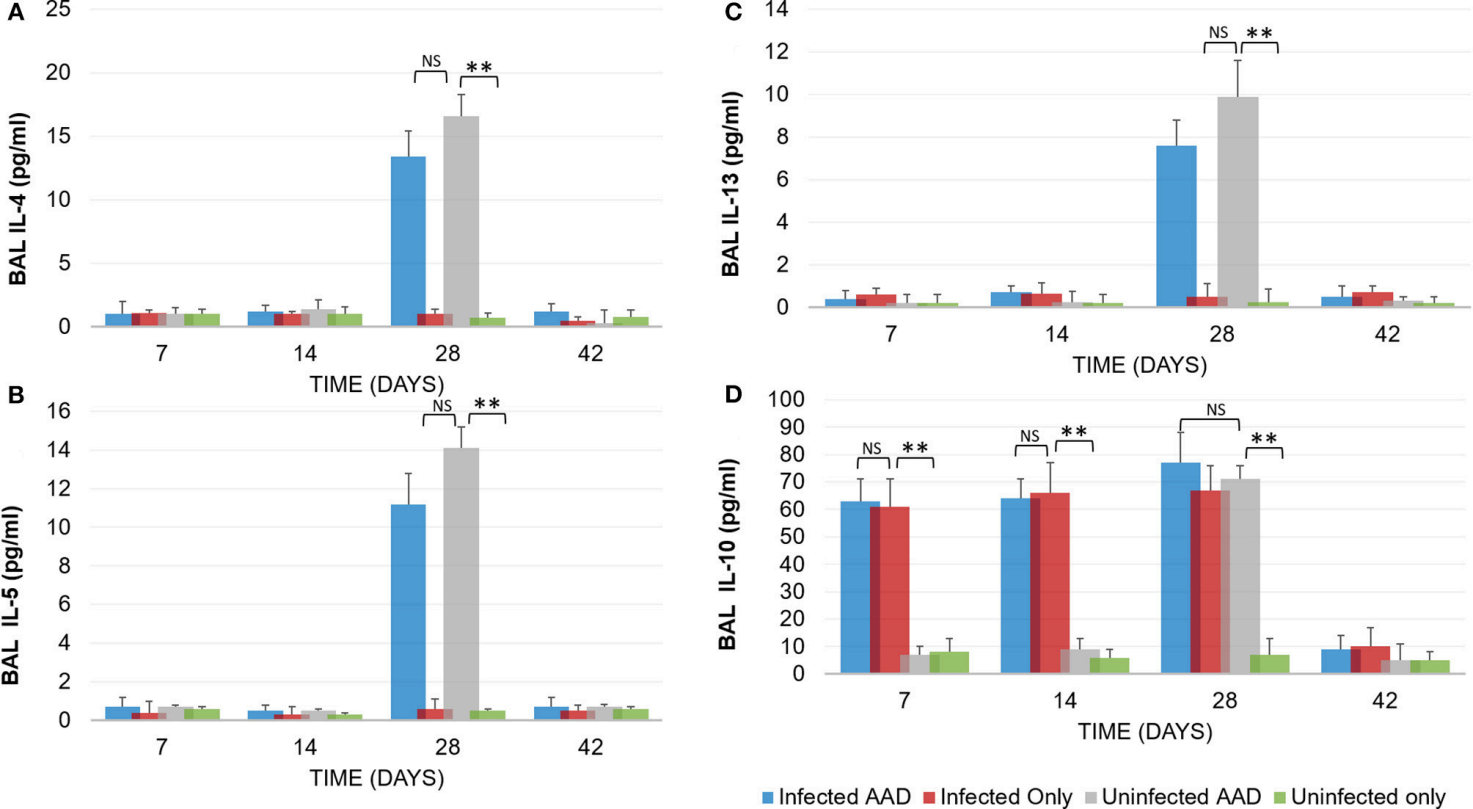
TH2 Cytokine production by AAD induced mice. Following the induction of AAD using Ova as described in the methods, both infected and uninfected animals (*N* = 5 animals per group) produced elevated amounts of IL-4, IL-5 and IL-13 **(A–C)**. Infected animals produced IL-10 throughout the treatment period until clearance of the organism from the lungs **(D)**. Uninfected animals with AAD induced also produced elevated levels of IL-10. All cytokine levels declined by day 42 when *Chlamydia* was cleared from the lung tissue. **P < 0.0001 for uninfected controls compared to infected animals; NS represents a *p*-value that is not significant. There was no significant difference in cytokine levels between infected AAD and uninfected AAD animals for these Th2 cytokines. Results are representative of 2 independent experiments.

**Figure 5:**
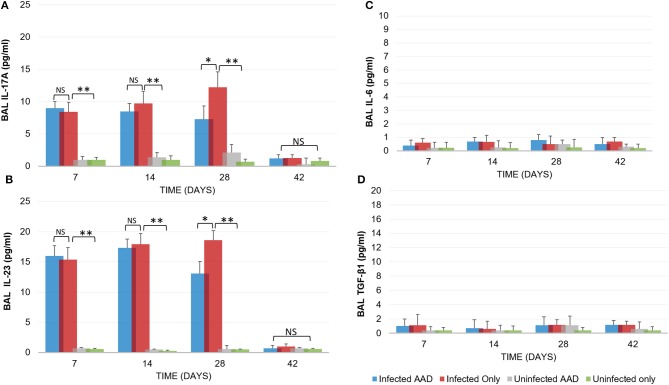
Inflammatory cytokine production. Infected animals displayed a moderate Th17 cytokine response (IL-17A and IL-23) in response to chlamydial infection **(A,B)**. However, no IL-6 production was seen in any animal group **(C)**. IL-17A and IL-23 levels diminished once chlamydial infections were cleared and TGF-β1 was not produced **(D)**. Uninfected and AAD induced only animals produced no Th17 or TGF-β1 cytokines. *N* = 5 animals for each treatment group. *P < 0.05, **P < 0.0001. *P*-value. An independent-sample *T*-test was performed for uninfected controls compared to infected animals; NS represents a *p*-value that is not significant. Results are representative of 2 independent experiments.

Animal models of allergic airways diseases (AAD) have been widely used to recapitulate the pathophysiology associated with recurrent exposure to allergens seen in human asthma(24). We utilized the common ovalbumin sensitization and challenge model here. The common features of this model is production and release of Th2 cytokines as well as eosinophilic and lymphocytic infiltration in the lung(24). *Chlamydia* infection typically induces a Th1 immune response, characterized by IFN-γ secretion and macrophage activation, which are important and necessary for clearance of this intracellular bacteria. In contrast, AAD is a Th2-driven disease characterized by the influx of Th2 cytokines, eosinophils, and allergic hyperresponsiveness. In this study, we characterized lung infection with *Chlamydia*. BAL fluid was analyzed for the presence of Th1 and Th2 cytokines using commercial cytokine kits as instructed by the manufacturer. In this model, following respiratory infection with *Chlamydia*, mice were sensitized to Ova and some groups were challenged over several days while others remained unchallenged for comparison as controls (Table 1). The data confirmed that infected animals secreted significantly elevated amounts of primarily Th1 cytokines (IFN-γ, TNF-α, IL-2, and IL-12) compared to uninfected animals (*P* < 0.001; Figures 3A–D). Th1 cytokine levels peaked on days 14 and 28 and dropped off by day 42 once respiratory infections had been cleared. Animals that were AAD induced responded to ovalbumin with a robust Th2 cytokine response (IL-4, IL-5, and IL-13) compared to their un-induced counterparts (*P* < 0.001; Figures 4A–D). Th17 cytokines (IL-17A, IL-23) were also elevated and peaked at day 14 in infected animals (*P* < 0.001; Figures 5A,B). Uninfected AAD induced animals had negligible levels of Th1 and Th17 cytokine production. IL-10 levels (Figure 4D) increased in infected and AAD induced animals, while TGF-β1 and IL-6 (Figures 5C,D) were not produced in significant quantities in any group of animals examined. It is important to note here that IL-17A is a potent neutrophil recruiter to the airways (25).

**Table 1:** Animal grouping and treatments.

**Adult Mouse Groups**	**Treatment Description**
Group 1	Infected; Allergic Airway Disease Induced (day 23)
Group 2	Infected
Group 3	Uninfected; Allergic Airway Disease Induced (day 23)
Group 4	Uninfected

*Two week-old Balb/C mice, grouped based on infection and treatment. Each group contained 15 animals and allergic airway disease was induced using ovalbumin as described in the methods. Group 2 animals were infected but had no AAD induced, while group 4 animals were not infected and had no AAD*.

**Figure 6:**
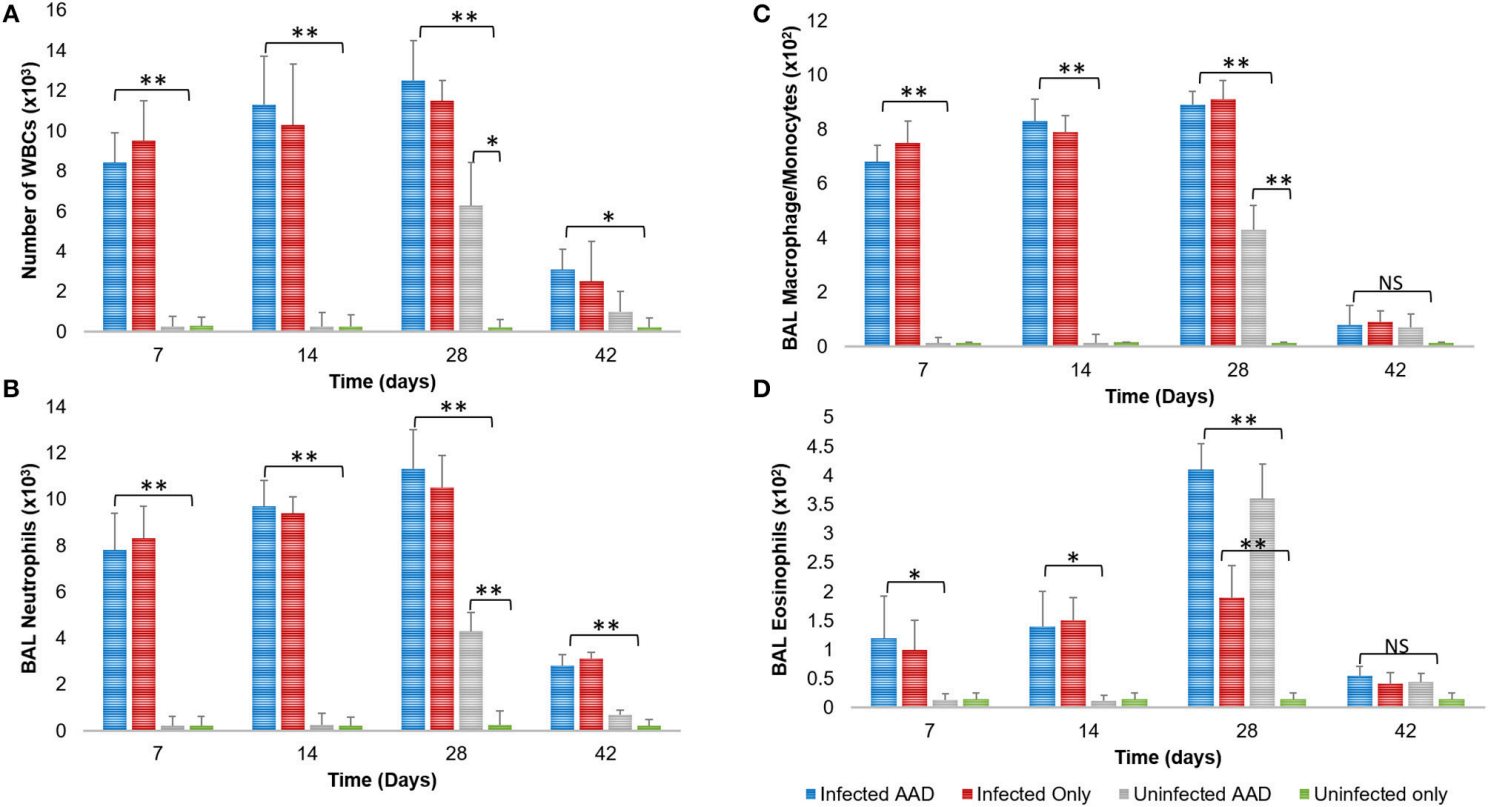
BAL cellular response to infection and AAD Induction. Infected groups with active infections had elevated levels of total WBCs, neutrophils, and macrophages compared to all other groups, which peaked on day 28 post infection **(A–C)**. Animals displayed significantly elevated numbers of BAL eosinophils on day 28 when AAD was fully induced **(D)**. All cell counts decreased to uninfected levels after infections were cleared and AAD subsided. *N* = 5 animals for each treatment group. *P < 0.05, **P < 0.0001. *P*-value. An independent-sample *T*-test was performed for uninfected controls compared to infected animals; NS represents a *p*-value that is not significant. Results are representative of 2 independent experiments.

The robust cytokine response in the airway of mice that were only *Chlamydia*-infected (Group 2) was complemented by the accumulation of predominantly macrophages (9.0 × 10^2^) and neutrophils (9.9 × 10^3^) in the BAL fluid of infected mice 28 days post-infection (Figures 6A–C), along with markedly less eosinophils (1.9 × 10^2^; ^*^P < 0.05, ^**^P < 0.0001; Figure 6D). In contrast, 28 days pi AAD induced animals presented with significantly elevated eosinophils (3.6 × 10^2^) compared to un-induced mice (Figure 6D). Mice that were both AAD induced and infected had elevated levels of all cell types 28 days post-infection, compared to AAD only groups (^*^P < 0.05, ^**^P < 0.0001), except for eosinophils and macrophages at day 42.

### Assessment of hepoxilin production in lung tissue

**Figure 7:**
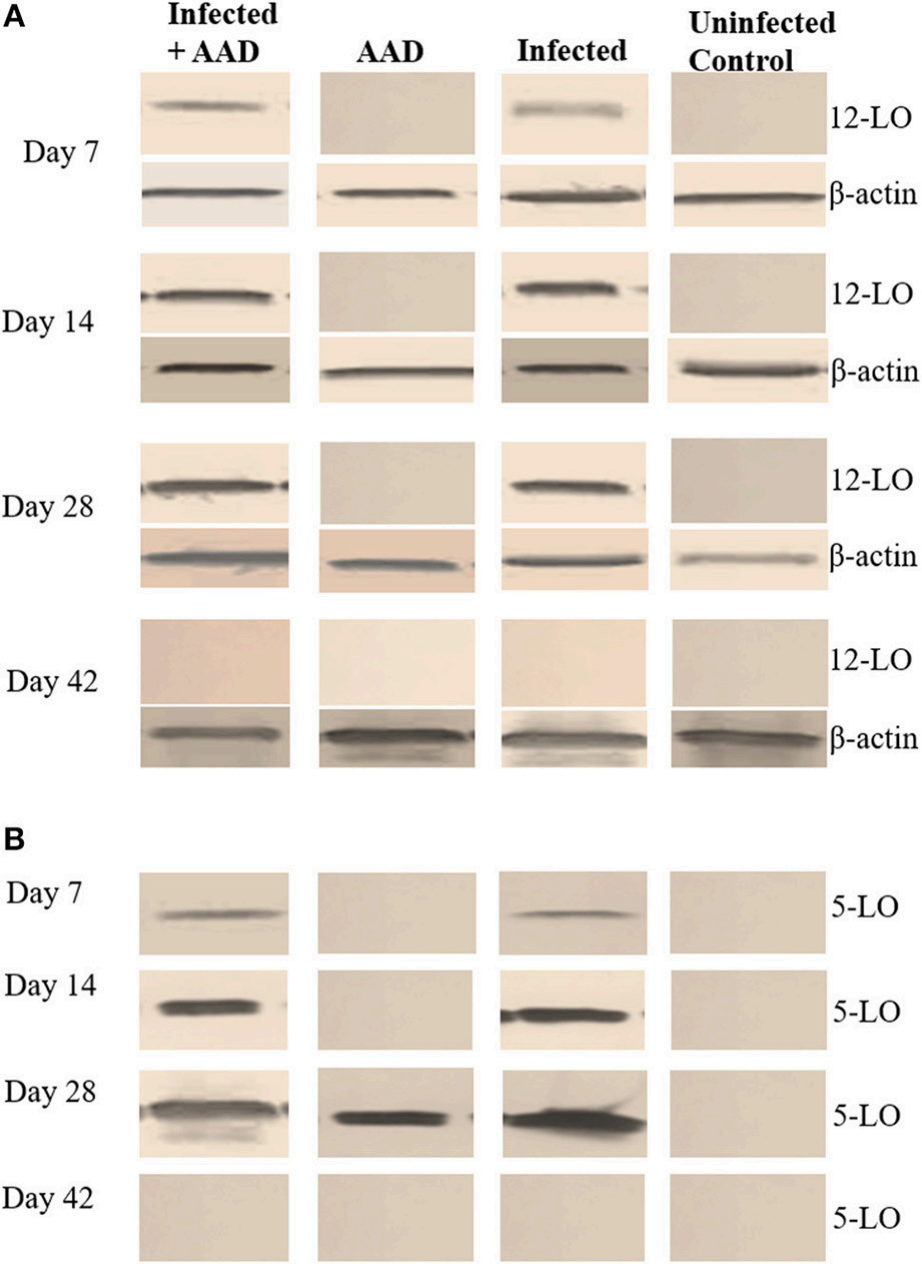
5-LO and 12-LO Western Blot of mouse lung tissue. The12-LO (76kDa) protein was present in infected lung tissue from days 7 to 28. However, it undetectable in the AAD only and uninfected animals **(A)**. 5-LO (79kDa) protein was detected in the lung tissue of infected animals on days 7 through 28, as well as AAD induced animals on day 28 following induction of AAD with Ova **(B)**.

In order to confirm the presence of hepoxilin in the lung tissue, we utilized Western blotting with specific antibodies to detect a hepA3 precursor, 12 lipoxygenase. We also sought to determine the presence of 5-lipoxygenase, which is a leukotriene precursor. Hepoxilin (HXA_3_) itself has an extremely short half-life so direct detection is difficult (21). Western blot revealed that 12-LO was present in infected mice as early as day 7 post-infection and remained detectable until day 28 (Figure 7A). However, 12-LO was not detected in uninfected animals or animals induced for AAD but were not infected. The leukotriene precursor enzyme 5-LO, was detected in the lungs of infected mice starting on day 7. It was only seen on day 28 in the lungs of mice with AAD induced (Figure 7B).

**Figure 8:**
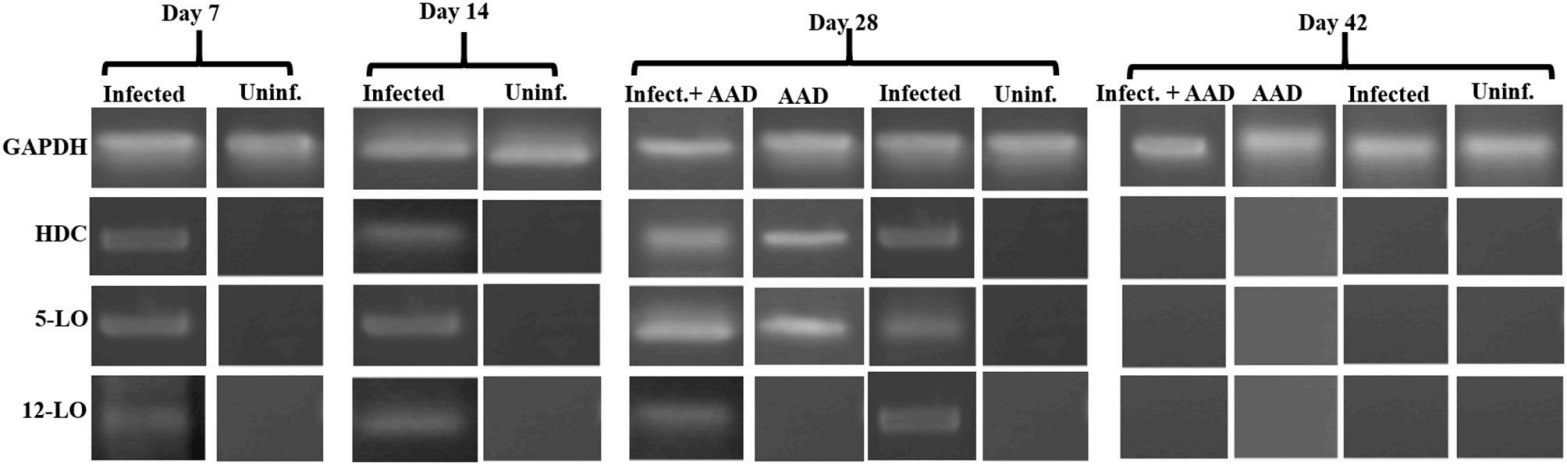
5-LO, 12-LO, and HDC mRNA expression in mouse lung tissue. Analysis of mouse lung tissue for the mRNA expression of 5-LO, 12-LO, and HDC revealed that only infected groups express 12-LO from days 7 to 28. 5-LO was expressed in both infected groups on days 7 through 28 as well as AAD induced groups (only on day 28). By day 42 there was no expression of any of these genes, reflecting infection clearance and relief from AAD induction.

In addition, we examined the lung tissue for HDC, 5-LO, and 12-LO mRNA expression, using RT-PCR. The data supports our Western Blotting results. 12-LO mRNA was upregulated only in infected animals, and undetectable in AAD or uninfected animals (Figure 8), while 5-LO expression was upregulated in infected animals and AAD induced groups (Figure 8). Interestingly, HDC mRNA was upregulated as expected in AAD induced groups, but was also detected in infected animals which were not AAD induced (Figure 8). Expression of 5-LO and 12-LO were seen as early as day 7 until day 28, while HDC mRNA was present in infected mouse groups from days 7 to 28, but was not seen in AAD only groups until day 28, following AAD induction. No expression as seen at day 42 post-infection (Figure 8). Our results suggest that respiratory tract chlamydial infection alone is sufficient to induce 12-LO expression (ultimately hepoxilin) as well as induce HDC expression (histamine) in lung tissue.

### Assessment of histamine production in BAL neutrophils

**Figure 9:**
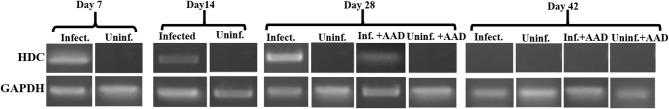
HDC mRNA expression in BAL neutrophils. Analysis of purified BAL neutrophils for the mRNA expression of HDC confirmed only infected animals expressed HDC from days 7 to 28. HDC was not present in BAL neutrophils recovered from the airways of AAD only or uninfected groups. HDC expression ceased in these cells after bacterial clearance.

**Figure 10:**
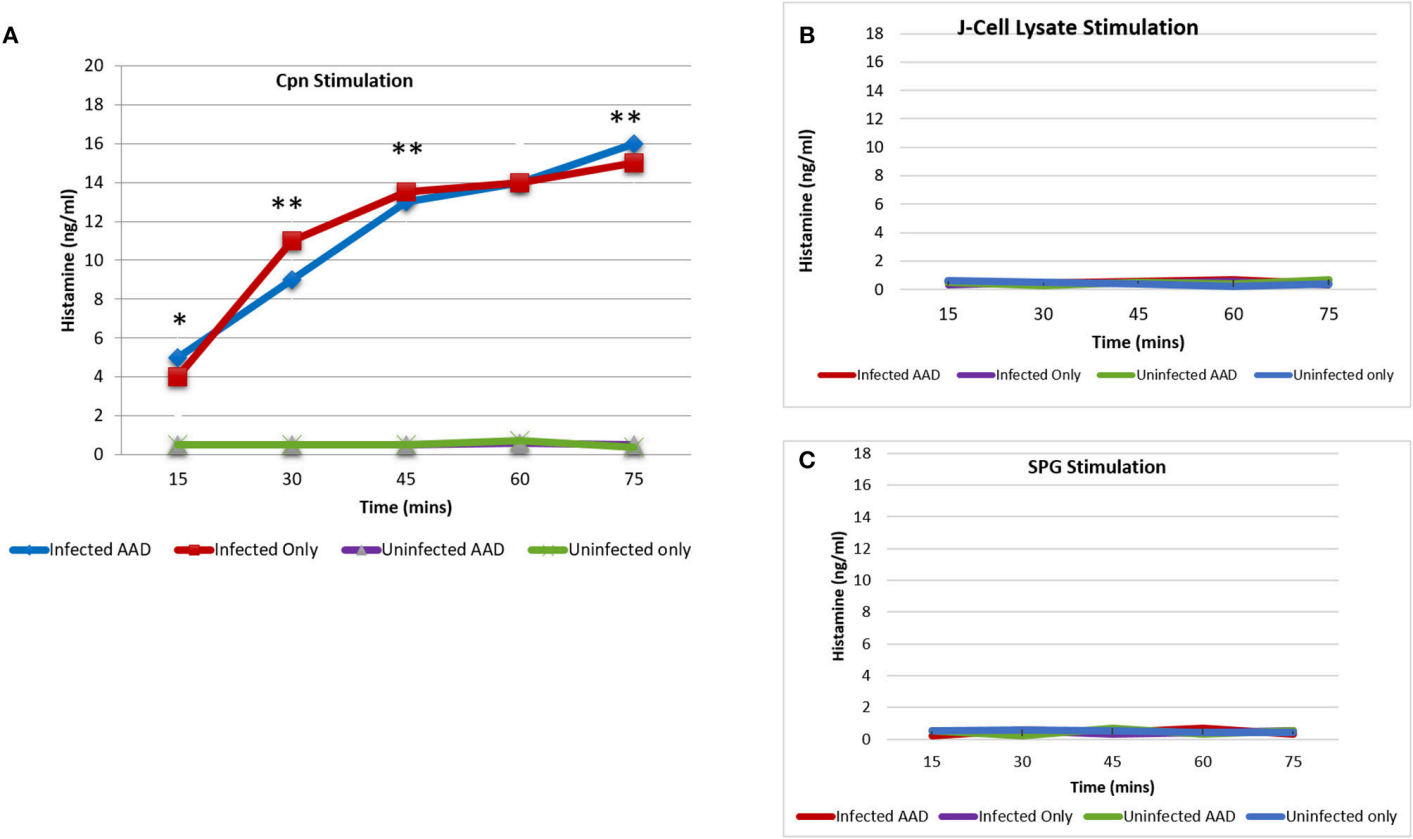
Histamine release from BAL neutrophils in response to chlamydial antigen. BAL neutrophils isolated at day 28 post-infection, were challenged with chlamydial EB antigens *in vitro*. Subsequent histamine release into the supernatant was assessed using ELISA. BAL neutrophils from infected animals released significant amounts of histamine in response to chlamydial antigen challenge. Histamine levels peaked 75 min post-stimulation **(A)**. Neutrophils from uninfected animals had no release of histamine in response to chlamydial stimulation **(A)**. Histamine release was undetectable in any group, in response to stimulation with SPG or J774A.1 cell lysate proteins **(B,C)**. The data confirms the requirement for *Chlamydia* sensitization by the airway neutrophils for histamine production and subsequent release. Each point on the curve represents the mean ± SEM. Data were analyzed with a paired Student *t*-test.*P < 0.05; **P < 0.001.

Histamine is an important mediator in allergic reactions. However, only a small subset of cell types have been shown to produce it. Recent studies have clearly shown that human neutrophils are legitimate histamine producers. The researchers went on to show that antigens to which patients with allergies were sensitized stimulated release of histamine from neutrophils (18). In order to determine if chlamydial infection could induce histamine production in neutrophils, BAL neutrophils were isolated and purified as described in the methods section and assessed for mRNA expression of HDC using RT-PCR. Our data demonstrated that neutrophils isolated from infected groups, regardless of AAD induction, had significant expression of HDC from day 7 to 28 (Figure 9). Neutrophils from AAD induced animals and uninfected animals had no expression of HDC, even at day 28 when AAD was fully induced. By day 42, HDC was no longer detected as a result of clearance of infection and alleviation of AAD (Figure 9). In addition to examining mRNA expression of HDC, purified BAL neutrophils were stimulated *in vitro* with heat killed chlamydial elementary bodies. The cells were then assessed to determine if this treatment elicited the release of histamine into the supernatant. Neutrophils from *Chlamydia*-infected animals released histamine in response to stimulation with chlamydial antigen (Figure 10A). Histamine levels increased over time in response to antigen stimulation, and peaked 72 minutes post-exposure. Stimulation of BAL neutrophils with SPG or J774A.1 macrophage protein lysate did not induce histamine release in any group (Figure 10). Our data demonstrate that chlamydial infection induces histamine production in BAL neutrophils. Furthermore, direct stimulation of these cells with chlamydial antigen resulted in subsequent histamine release.

## Discussion

In addition to well-defined environmental causes, accumulating evidence suggests that respiratory tract infections play an important role in the pathogenesis of asthma (26–28). Among these infections, *C. pneumoniae* appears to be a cause of exacerbation as well as a possible initiator in the development of chronic asthma (29). Clinical studies have shown that early-life *C. pneumoniae* infection is associated with increased incidence of childhood asthma (12, 13). Furthermore, this asthmatic phenotype is characterized by infiltration of neutrophils, a phenomenon not seen in typical allergen-induced airway hyperactivity, which is predominated by eosinophils and basophils (23). Identifying host response factors that may contribute to the pathogenesis of infection-mediated airway hyperactivity (AHA) is critical for establishing a functional association between *Chlamydia-*induced airway inflammation and the subsequent development of appropriate interventions. Surprisingly, recent reports have found that almost 50% of persistent asthma cases are accompanied by an increase in airway neutrophils and these patients were not only more refractory to corticosteroid treatment, but represented the subset of asthmatics at greatest risk for asthma-related mortality(30).

A neutrophilic airway phenotype has long been associated with hard-to-control asthma that is often steroid-resistant (30). Beyond the clear inflammatory response caused by the presence of neutrophils, we also wanted to determine if these neutrophils produce histamine in response to *Chlamydia* infection. A previous study published by Xu et al. demonstrated that *Mycoplasma pneumoniae*, a prevalent respiratory pathogen that is commonly co-isolated with *C. pneumoniae*, provokes histamine release from neutrophils in the murine airways (23). One of the most significant findings from the current study is that neutrophils from *Chlamydia*-infected murine airways were highly enriched in histamine compared with naive neutrophils. These neutrophils had significantly upregulated mRNA encoding histidine decarboxylase, the rate-limiting enzyme in histamine synthesis. However, it is still not clear which metabolite(s) or inflammatory mediators represent the major neutrophil chemoattractant(s) in the infected airways of asthmatics.

Hepoxilins are biologically active metabolites of arachidonic acid formed through the 12-lipoxygenase pathway. Hepoxilin A3 has been implicated as a key regulator of mucosal inflammation because it is produced by mucosal epithelial cells and functions as a guide to neutrophils to cross the epithelial tight junctions through the establishment of a gradient(22). Hepoxilins have been identified as potent neutrophil chemoattractants in the intestinal mucosa, playing an even greater role than IL-8 (22). One of the goals in this study was to determine if *Chlamydia* infection of the airways induce the release of hepoxilins, thereby enhancing neutrophil migration to the airways. We hypothesized that neutrophils, provoked by *Chlamydia*-induced lung infection, undergo transmigration through an IL-8/hepoxilin gradient and greatly expand their capacity to synthesize histamine, thereby contributing to airway inflammation and pathology. Our data suggest that chlamydial infection induces a Th1 type cytokine response with corresponding macrophage and neutrophil infiltration. A better understanding of neutrophil recruitment across the respiratory mucosa in infection-mediated asthma, may inform the development of novel therapeutic strategies for the treatment of severe asthma patients. When AAD was induced, a mixed Th1/Th2 cytokine and cellular response was seen in BAL fluid and lung tissue of infected animals, while uninfected AAD controls responded with a robust Th2 cytokine profile. These results are similar to those previously reported by Horvat et al. (29, 31) using a similar Ova mouse model. In addition, our current study found that infected animals also produced moderate levels of Th17 cytokines (IL-17A, IL-23).

Lung tissues were examined for the presence of hepoxilin (through the detection of 12-lipoxygenase), confirming that only *Chlamydia-*infected mice expressed 12-LO protein and mRNA. Animals in which we only induced AAD or ones that were uninfected did not produce detectable amounts of 12-LO. These data suggest that respiratory *Chlamydia* infection induces the production of 12-LO as early as 7 days post-infection and strongly suggests that hepoxilin is a significant player, which could be acting as a chemoattractant for airway neutrophil infiltration. In addition, we demonstrated that chlamydial infection was sufficient to elicit mRNA expression of HDC, indicating the production of histamine within the lung, which most likely contributes to airway hyperresponsiveness.

We also examined BAL neutrophils for the ability to produce histamine. We found that animals with AAD induced, elicited neutrophil infiltration to the lung, but these cells did not express HDC. In contrast, infected animals produced significant amounts of neutrophil, regardless of AAD and more interestingly, that these neutrophils in fact produced histamine in response to chlamydial antigen exposure. We observed HDC upregulation in BAL neutrophils by day 7, which was sustained until the infection was resolved. Furthermore, upon stimulation of BAL neutrophils with chlamydial antigen caused histamine degranulation from these cells. This data provides a mechanism as to how neutrophils can mediate sudden and severe asthma airway hyperresponsiveness. Together this provides us insight into how neutrophils can cause airway hyperresponsiveness as seen in asthma.

Overall, this data demonstrate that respiratory chlamydial infection induces a mixed Th1/Th17 cytokine response, characterized by significant neutrophil infiltration. Induction of AAD in the presence of this infection adds Th2 cytokines to the milieu. Neutrophil infiltration appears to be, at least, partially mediated by the production and secretion of hepoxilin across the respiratory mucosal surface. However, this study could not definitively prove that hepoxilin is the major chemoattractant here. Further studies exploring the arachidonic acid pathway activation in chlamydial lung infection should shed more light on the exact pathways involved. Of significance is the data showing that the population of neutrophils infiltrating the lung tissue in response to chlamydial infection produce and release histamine. Therefore, in addition to regular neutrophil-mediated inflammation, neutrophils could contribute to airway hyperresponsiveness through histamine release in response to the presence of chlamydial antigens. We have therefore provided a novel mechanism for airway hyperresponsiveness and pathology in infection mediated asthmatic initiation or exacerbation. Since neutrophils are one of the first cell types recruited to the site of an allergic reaction, they may also influence clinical presentation and play a role in the development of severe chronic asthma and the onset of sudden severe attacks in the presence of *C. pneumoniae*. Our data suggest that if hepoxilin is released in response to airway infection, pharmacologic regulation of airway hepoxilin should provide a novel therapeutic strategy. This is especially important for a growing subset of chronic asthmatics who are refractory to maximal doses of inhaled corticosteroid and are at increased risk for asthma mortality.

## Author contributions

KP performed all the experiments leading to this manuscript. He also assisted with drafting the methods section and creating figures. WW initiated this study and wrote the first draft of the manuscript. He helped perform data analysis and creating figures and graphs. Both authors contributed to and approved the final version of this manuscript.

### Conflict of interest statement

The authors declare that the research was conducted in the absence of any commercial or financial relationships that could be construed as a potential conflict of interest.
